# The North American Review

**Published:** 1881-04

**Authors:** 


The North American Review.—Is unquestionably one of the most valuable publications in this country in every respect. Both sides of any proper question may be fully discussed, *pro* and *con*, in its pages without prejudice or partiality. We call attention elsewhere to the general contents of a recent number, as an illustration of what it is doing. It should be in the hands of every thinker in the land.

